# The Voice of Endo: Leveraging Speech for an Intelligent System That Can Forecast Illness Flare-ups

**DOI:** 10.1145/3706598.3714040

**Published:** 2025-04-25

**Authors:** Adrienne Pichon, Jessica R. Blumberg, Lena Mamykina, Noémie Elhadad

**Affiliations:** Columbia University, Department of Biomedical Informatics, New York, NY, USA; University of Delaware, Newark, DE, USA; Columbia University, Department of Biomedical Informatics, New York, NY, USA; Columbia University, Department of Biomedical Informatics, New York, NY, USA

**Keywords:** chronic illness, forecasting, voice analysis

## Abstract

Managing complex chronic illness is challenging due to its unpredictability. This paper explores the potential of voice for automated flare-up forecasts. We conducted a six-week speculative design study with individuals with endometriosis, tasking participants to submit daily voice recordings and symptom logs. Through focus groups, we elicited their experiences with voice capture and perceptions of its usefulness in forecasting flare-ups. Participants were enthusiastic and intrigued at the potential of flare-up forecasts through the analysis of their voice. They highlighted imagined benefits from the experience of recording in supporting emotional aspects of illness and validating both day-to-day and overall illness experiences. Participants reported that their recordings revolved around their endometriosis, suggesting that the recordings’ content could further inform forecasting. We discuss potential opportunities and challenges in leveraging the voice as a data modality in human-centered AI tools that support individuals with complex chronic conditions.

## 1 Introduction

Chronic illnesses are challenging for care systems and individuals to manage. These conditions are often heterogeneous in their symptoms and presentation, which means that individuals need personalized support. In some chronic conditions, management is further complicated by unpredictability of flare-ups and uncertainty in how to alleviate them. Endometriosis, a systemic, painful condition with a debilitating impact, is an example of such complex illnesses [4, 83, 110].

Evidence shows that people living with endometriosis want to monitor their illness day-to-day to ascertain their health status and illness journey, but also to anticipate flare-ups. However, identifying potential triggers and even assessing variations in health status reveals to be particularly difficult for patients [83]; thus, both detecting current health status and forecasting future events are complex tasks for patients. In endometriosis, there is no biomarker to date that can diagnose the condition, let alone help patients monitor their day-to-day health variations [4].

There is a rich tradition within HCI of creating personal informatics systems designed to support patients in monitoring and making sense of their own data and experience of illness, as well as guide them in the tasks of self-management and decision making [31, 34, 61]. Personal informatics apps designed for various diseases — *Trackly* for multiple sclerosis [7], *BlueStar* for type 2 diabetes [3], and *Jointfully* for osteoarthritis [45] — have encouraged participation in health-promoting behaviors.

For a complex chronic condition, providing personalized health status forecasts has significant potential for improving patient quality of life, allowing people to make informed decisions about the management of their condition. In machine learning (ML), a prediction is the output generated by a trained model that the model infers when processing new, unseen input data based on learned patterns — detection can be considered the prediction of past or current outcomes, while a forecast is a specific type of prediction focused on inferring future values based on historical data and patterns. Forecasting is commonly used for various day-to-day applications, e.g., weather and traffic, and is helpful for planning ahead in these contexts [10, 40]. Yet, few studies have investigated the use of forecasts to support patients, especially in a disease such as endometriosis with so much heterogeneity and uncertainty. There is an opportunity for generating personalized health forecasts, which have the potential to mitigate the impact of flare-ups on daily life.

Forecasting such flare-ups automatically would be greatly facilitated by biomarkers that fluctuate along with day-to-day variations of disease experience. However, in many diseases such as endometriosis, it is not always known what, if any, biomarkers are amenable to disease monitoring. Passively collected and naturally occurring data sources, coupled with appropriate analysis techniques, have the potential to yield illness insights [5, 78].

In the case of an inflammatory condition like endometriosis, markers of inflammation may take the place of biomarkers for the sake of flare-up detection and forecasting. But tracking such markers daily is not always feasible for patients. There is a lot of research for using voice as an interaction mechanism or journal to log experiences [62, 65, 69, 82, 84, 85]. Some existing research suggests that speech and voice signal can be used to detect physiological well-being in the long term [46, 50, 106]. But little is known about using voice as a meaningful data modality for automated health forecasting.

Artificial Intelligence (AI) techniques are needed for developing such forecasting abilities, and beyond the need to develop such technology, there is also a need to design an intelligent system that would leverage this forecasting functionality. In this paper, we take a human-centered approach in informing the design of such a tool. We investigate patients’ acceptability and perceived benefits and challenges of an imagined AI tool which tracks their health and forecasts their flare-ups through the analysis of their voice. We work with patients to imagine this tool and speculate about speech as a data source that could reflect and forecast underlying health status.

Our Research Questions include **(1)** What is the patient experience and potential impact of using voice as a modality for data collection? **(2)** What are the potential benefits and drawbacks, as perceived by patients, of an intelligent system that could provide forecasts for upcoming symptom flare-ups in the context of complex chronic illness? **(3)** Specifically using the voice, what are the potential benefits and drawbacks, as perceived by patients, of an intelligent system that could provide forecasts for upcoming symptom flare-ups in the context of complex chronic illness? To explore these questions, we engaged individuals with endometriosis in a speculative design activity lasting six weeks — we asked participants to submit voice recordings and symptom logs as if using an intelligent system for flare-up forecasts, but we did not provide forecasts. The aim was to situate individuals in the same context that they would use such a tool, so that they could be in a similar mindset and be prepared to ideate with us at the end of the study, based on their real-world experiences. At the conclusion of this activity, we brought participants together in a focus group setting to reflect on how voice tracking may have factored into their real-world experiences. This study has the following key contributions:

- Our research identified a potential emotional benefit for patients to recording their voice.
- Our research identified that an imagined intelligent system leveraging voice analysis might validate patients in their experience of disease, help them advocate for themselves in clinical encounters, and combat the normalization of their symptoms.
- We contribute key insights for how to move forward in developing interactive AI systems to support individuals with complex chronic illnesses that remain human-centered, enhance control, and promote trust.

## 2 Background and Related Literature

### 2.1 Endometriosis

Endometriosis is a chronic condition that impacts approximately 10% of people assigned female sex at birth. It is an inflammatory disease that is characterized by chronic pelvic pain, sub-fertility, and a number of other symptoms, such as fatigue, bloating, and pain across multiple body locations [28, 33, 38, 99]. Along with this wide range of possible symptoms, the illness experiences of people with endometriosis vary greatly: while some people may be asymptomatic day to day, many experience debilitating pain that varies in type, pattern, and intensity across individuals [17, 103]. Some patients experience cyclical manifestations of symptoms while others experience fully unpredictable flare-ups. The complexities, irregularities, and unpredictable nature of symptoms contribute to the negative impacts on all dimensions of health [75].

Flare-ups of endometriosis, defined qualitatively as a dramatic increase in symptom frequency and intensity over a day or longer, significantly disrupt individuals’ lives and force them to rearrange their routines around symptom onset. As one patient described, *“It becomes its own full-time job”* to track, manage, and mitigate flare-ups [83]. Beyond the challenges of managing flare-ups, the uncertainty of when they will occur presents a significant additional burden.

Researchers in endometriosis have focused on identifying biomarkers indicative of the presence of disease, but so far to no avail. There are no known biomarkers for the long-term monitoring of the disease, the way A1C does for diabetes for instance, and there are no known biomarkers that can help monitor day-to-day variations in health status, the way glucose measurements after a meal for instance does [4].

Research in HCI highlights that personal informatics tools can support patients in coping with chronic illness. Such tools have the potential to support a range of patient needs, including to enable individualized insights and provide personalized recommendations for management [31, 61]. Phendo is a personal informatics tool for self-tracking the lived experiences of endometriosis, developed through participatory research with end-users, that is part of a broader citizen science initiative to address gaps in medical knowledge about the disease [71, 72].

### 2.2 Speech and voice technologies in the wild

Intelligent systems that use speech as an interactive mechanism have long been studied in HCI and have become ubiquitous in real-world technologies for over a decade. Speech and voice are leveraged in multiple ways in interactive technologies. Some systems talk to users, but do not capture the user’s voice (e.g., an audio guide for blind museum visitors [57]). Other systems capture the user’s speech but do not talk back to users, and analyses focus on the content of users utterances, rather than the patterns of voice signals (e.g., voice to text [93] and voice to emoji [108]). Finally, some technologies use speech as an interactive communication modality, both capturing the content of users’ speech and uttering output back to the user in the form of a dialogue (e.g., Google Assistant for supporting a cooking task [37, 52]). Embodied AI, including voice assistants, conversational agents, and chatbots, have become increasingly popular in consumer technologies (e.g., Alexa and Siri), suggesting widespread comfort with voice-based interactions. These tools provide a human-like conversational experience and are particularly valuable for helping users complete tasks when their hands or eyes are occupied [20, 58, 73, 86].

Although voice-user interfaces are commonly used for simple, user-initiated tasks like playing music, retrieving web-based information, and controlling smart home devices, they are increasingly being explored for applications in well-being and healthcare settings. For example, interacting with the social robot PARO was found to improve mood and inspire positive feelings that helped alleviate pain [87]. In the context of smoking cessation, individuals who engaged with automated phone calls using interactive voice response technology reported higher abstinence rates, attributing this success to the social support provided by the system [91]. Voice-based systems have also been developed to assist older adults and individuals with low vision or literacy [16, 94]. Furthermore, research highlights both opportunities as well as risks of disruption of using voice data to supplement documentation of patient visits in clinical settings [107].

### 2.3 Self-reporting, passive tracking, and naturally occurring behaviors to capture useful information

#### Active tracking and self-reports.

Research in HCI encourages a range of methods for capturing user experiences, which can promote reflection and accurate data capture. One approach is Ecological Momentary Assessment (EMA), which enables real-time data capture in natural settings with minimal intrusiveness. EMA research guides designers to ensure technologies are adaptable to users’ daily lives to reduce burden while promoting meaningful and accurate data collection [26]. While there are benefits to self-reports, such as collecting valuable user-centered representations, there are also challenges in collecting data that accurately and consistently represent users’ experiences [27]. Memory recall and emotional processing impact how individuals document health-related events, and interactions with digital tools can shape reflection and emotional engagement [66]. Karapanos et al. documents the fluidity of memories, suggesting that memories are not static and thus retrospective self-reports can be influenced by these changes [54]. To address some of these limitations, Li et al. introduced *DiaryHelper* to scaffold users’ journaling and support them in capturing rich, contextualized data by improving recall, supporting multi-modal tracking, and reducing burden on users [62].

Multimedia data — such as photographs, videos, and voice recordings — can complement structured data by capturing real-world experiences along with their surrounding contexts. Research has demonstrated their value in recording health-related data, enhancing recall, and promoting self-reflection [85]. For example, incorporating photos into manual self-tracking for food journaling can provide useful context, enhance recall, and promote mindfulness and reflection that can enable users to focus on a range of health-related goals [23]. Photo-based journaling that combines in-the-moment reflection with retrospective reflection has benefits to contextualized and meaningful data capture [55]. Beyond photos, videos also offer a flexible medium for creatively documenting and reflecting on daily activities, symptoms, and overall health status [65] and can facilitate introspection, allowing users to construct rich, holistic narratives of their health experiences rather than relying on fragmented symptom checklists [69]. Voice-mail studies can be a flexible method for capturing data in real-time and under naturalistic conditions that can overcome some of the challenges and intrusiveness of traditional paper-based diary approaches [82]. Thus, tracking health data with audio diaries is a patient-centered and inclusive approach that can represent the nuanced contexts where tracking occurs, allowing for holistic capture of behaviors over time and facilitating meaningful insights [84]. These data modalities also hold social value by fostering connection and empathy — sharing expressive, self-reflective content with family, friends, or others experiencing similar conditions can promote empowerment, emotional well-being, and a sense of togetherness, which also supports personal health priorities and goals [44, 65, 80].

#### Wearables and sensors.

While actively self-tracking illness data can provide useful information for personal informatics tools, it can also impose a significant burden on users. Passive tracking and other low-burden data capture methods offer a promising alternative, reducing user effort while collecting meaningful health-related data and insights. Wearable consumer devices and other sensors have become increasingly pervasive, e.g., smartwatches capturing in-the-moment biometric data on physiological states and activity levels, including heart rate [64], movement and gait patterns [76], and skin conductance and temperature [2]. Less intrusive than traditional data collection methods, passive tracking enables continuous data capture, real-time insights, and timely, adaptable interventions [100]. It also offers opportunities for unobtrusive long-term health monitoring, complementing traditional diagnosis and management of chronic conditions [68]. Studies highlight their promise in diverse health-monitoring applications, including cardiovascular, activity, body temperature, and galvanic skin response systems, across a range of purposes including diagnosis and self-management [43, 92].

#### Voice and speech.

Just as wearables unobtrusively capture health-related data, features extracted from media such as photos, videos, and voice logs also have the potential to extract information that have the potential to be used as a type of biometric signal for an intelligent system. In particular, voice data extracted from recorded videos have been used to support health reflection and management. Given that prior research has documented clinical and social benefits of leveraging voice as a means of health documentation [18, 65, 85], it offers promise for capturing a user’s physiological state or health status. In fact, voice analysis has already been used to detect signals that support health assessment and management. Previous studies have identified the potential value of voice as a data source for detecting individualized well-being and physiological state [46]. For example, Huang et al. collected, transcribed, and extracted both auditory and contextual features from voice recordings, finding higher accuracy in detecting well-being compared to using traditional self-reported data [51]. Prior research has linked prosodic and acoustic features to the presence of chronic conditions such as depression [79, 106, 109], schizophrenia [13], Autism Spectrum Disorder [60, 97], and Parkinson’s Disease [6, 77], demonstrating that voice assessment can be used as a discrete screening method for underlying conditions and related health states. For example, both Zhao et al. and Wang et al. analyzed vocal acoustic characteristics to distinguish between patients with and without depression, suggesting potential uses for diagnosis and treatment [106, 109]. Similarly, Arora and Tsanas trained a support vector machine to differentiate between individuals with and without Parkinson’s Disease using voice recordings [6]. Voice data has also been used to assess coping strategies for breast cancer patients [104]. While many studies focus on emotional expression, they provide valuable insight for the potential of computational methods to study other disorders or detect aspects of speaker state, such as bodily inflammation [46].

### 2.4 Interactive systems to guide personal actions in illness management

Personal informatics tools help individuals understand their illnesses and guide health-related actions through analyses of historical and in-the-moment data. These systems enable users to gain insights into areas like sleep, food intake, and mood, then translate these into behavioral changes via self-reflection, personalized feedback, and recommended actions [11]. HCI has a rich history of designing technologies to support reflection that can foster wellbeing [12] and to enable the sensemaking process so that users can identify information that is useful for health and wellness, derive insights to inform health behaviors, and carry out health actions that account for these new personalized insights [70]. A variety of personal informatics systems that capture multi-modal information to generate personalized, health-related feedback have been developed [11, 23, 55, 88]. Designing for a range of self-tracking and self-reflection mechanisms — such as creating new trackers [59], implementing user-specific recommendations, and involving partners in management [74] — has been documented as critical for supporting users [22], sustaining engagement [42], and promoting diverse, sometimes unexpected, goals [69].

Though in-the-moment health predictions are useful for guiding self-reflection and promoting well-being goals, only limited research exists on using health-related data for forecasting (future-oriented) as opposed to detection (past or present-oriented). Preliminary work shows promise in generating forecasts to guide future actions. For people with diabetes, Desai et al. found that incorporating personalized forecasts into glucose tracking and nutritional decision-making helped individuals reflect on and adjust lifestyle patterns, build a nuanced and dynamic understanding of their condition, and guide their future choices [25]. Hollis et al., found that mood forecasting improved daily mood, ratings of self-awareness, and reported effects of activity on health status [47]. Similar advancements in forecasting have been documented using biometric data sources. For example, Das Antar et al. used passively collected activity and pacing biometrics to forecast the physical functioning of users with multiple sclerosis, a promising first step in promoting timely interventions to improve quality of life [24]. Other sources of biometric data, including pulse and respiratory rates, have been used to monitor and forecast health states for chronic obstructive pulmonary disease [95].

Our study aims to investigate the potential use of forecasts to guide health-related actions and behaviors, while imagining the potential of leveraging the voice as a naturally occurring source of biometric data. As endometriosis is a complex chronic illness, the possibility of forecasting flare-up onset could allow individuals time to manage flare-ups proactively, instead of in-the-moment, potentially using the voice as a means of capturing relevant data that might be used to infer an individual’s future health status.

## 3 Methods

We conducted a study with individuals with endometriosis, where we engaged participants in a speculative design activity related to using voice analysis to forecast illness flare-ups. We asked participants to submit daily logs consisting of a short voice recording and a structured symptom checklist for six weeks. At the conclusion of the study, we conducted focus groups to understand the participants experience of voice recordings and their perspectives on the possibility of using these recordings as part of an intelligent system that could forecast illness flare-ups. This study design is a form of participatory speculative design, specifically collaboration that Farias et al. refer to as “generative reflection” [32]. As argued by Baumer [9], a speculative design process can be a key approach for human-centered algorithmic design. Results from this study will inform the iterative ideation process of creating an intelligent system for using voice as a modality for supporting management of complex chronic illness.

### Study Design.

We created surveys to characterize the sample (i.e., demographics in a pre-study survey) and to assess the potential acceptability of the imagined tool (i.e., technology evaluation measures in a post-study survey). For the voice logs, we decided to collect “natural” data (i.e., reflecting real-world situations, which provides the most authentic and rich source of information) rather than “semi-natural” data (i.e., where data capture is constrained, for example by reacting to prompts) or “simulated” data (i.e., scripted). Even though “natural” data is a more complex and difficult to control data type, it is also the most authentic for capturing voice data and user experiences; it is also the most realistic to what a future intelligent system would encounter [1]. Additionally, we felt that not restricting the logs to illness-related topics would enhance autonomy and privacy for users by giving them the option to not talk about sensitive topics aloud, yet would still provide the data needed for the analysis of vocal characteristics.

Participants were recruited on social media, primarily through the broader research project’s profile. Inclusion criteria: at least 18 years old, speak English as a first language, endometriosis diagnosis, endometriosis symptoms in the past four weeks, iPhone users (so that the differences in hardware did not impact future signal analysis), and comfortable using iPhone to record and upload a file.

Researchers met with participants virtually for an onboarding session to explain the purpose of the research and the speculative design activity (see Supplementary Material). Participants were asked to submit daily voice recordings that a future intelligent system might be able to use to forecast an upcoming flare-up, but they would not receive any forecasts or analysis of their data in this study. Researchers provided instructions for the voice logs: Record 1–2 minutes daily, using the iPhone microphone; talk about whatever you choose, speaking naturally without a script (not limited to speaking about endometriosis symptoms); minimize background noise and speak clearly; and try to submit voice logs around the same time every day (to control for stress and hormonal markers of inflammation like cortisol, which has normal fluctuations throughout the day). Individuals were asked to select a time to receive daily text message notification reminders, around the time they thought they would be likely to submit their logs. Participants used a personalized link to access the daily log survey, where they could submit their voice log first and then log their symptoms with a structured checklist (see Supplementary Material for the full daily log survey). In addition to the daily logs, we also collected demographics in a pre-study survey and technology evaluation measures — related to the imagined use of a voice-enabled forecasting tool — in a post-study survey, including constructs from the Unified Theory of Acceptance and Use of Technology (UTAUT) [105] and Human-Computer Trust (HCT) measures [67]. All constructs were measured on a scale of 1 to 5, with 5 being most positive. Participants used the Qualtrics survey platform to submit all study data.

The focus groups centered on understanding the user experience of submitting voice logs and the potential benefits and drawbacks of a hypothetical intelligent system that could provide forecasts of symptom flare-ups (increase in symptoms in the future), in general and also one that uses voice analysis for this purpose. First, the moderator asked participants to reflect on the experience of submitting the daily logs, including the logistics of recording voice logs, what they talked about, the emotional experience of logging, and barriers and facilitators to capturing the data. Then, the moderator asked participants to imagine that we are building an app that uses AI to detect that they are very likely to have a bad symptom day in the near future. The moderator asked about the circumstances where this type of information might be useful and probed about preferences for incorrect predictions (“would you rather it always predicts flares but gets some wrong, or misses some but is always right?”). Finally, the moderator asked participants to think about a hypothetical system where an AI would use their voice and possibly the content of their recording to forecast these upcoming bad endo days. The moderator asked questions about participants’ desired functionalities, usefulness, and privacy. The full focus group guide is available in the Supplementary Material.

Participants received up to $150 compensation for the study ($15 for the pre survey and post survey, $25 for the focus group, and $2 per log plus a $11 bonus if they submitted at least 30 logs). The study was approved by Columbia University’s IRB.

### Analysis.

This paper reports on the speculative design activity, and leaves the technical analysis of the voice logs to a separate manuscript. We provide descriptive statistics and engagement details about how users interacted with the daily logs. We also report qualitative results from the focus groups at the end of the 6-week study. The descriptive statistics and plots were created using R; the qualitative analysis was conducted using Excel.

We recorded the focus group discussions, then transcribed what was said among participants and checked the text against the audio recordings. Thematic analysis was guided by our goals to understand the real-world user experience and potential impacts of using voice as a modality for data collection, and to understand the benefits and drawbacks of using an AI-enabled tool to forecast flare-ups. We followed the methodology described by [15]. We coded the transcripts iteratively, starting with margin notes then grouping ideas together to search for and generate themes. We revised the codebook through discussions until we agreed that the data were represented in the proposed groupings. We defined the final themes and created descriptions for them, while also extracting quotes to illustrate how each theme relates to the research questions. Two authors coded the transcripts, with a Fleiss’ Kappa of 0.84 across all three transcripts [36]. This indicates very good agreement and high inter-rater reliability.

## 4 Results

In this section, we present the results of the speculative design activity. First, we give a brief overview of participants and engagement with submitting daily logs. Next, we present the technology evaluation measures from the post-study survey. Finally, we present the findings from the qualitative analysis of focus group data.

### 4.1 Speculative Design Activity

A total of N = 21 individuals with endometriosis enrolled in the study and were engaged in the speculative design activity. Participants represent diverse backgrounds and illness experiences. Demographics are presented in Table 1a, and menstrual and illness experience variables are presented in Table 1b. Over the six week study, participants submitted a total of 413 logs. Out of a maximum of 42 days, individual participants submitted an average of 20 (SD 11.4) daily logs, ranging from 0 to 39 (two participants did not submit any logs — one had unresolvable technical trouble, and one was lost-to-follow-up). Voice log data was successfully extracted from a total of 382 logs (some files were submitted in the wrong format, rendering the audio unavailable). The average duration of voice logs was 94 seconds (SD 41.4), ranging from 30 to 282 seconds. Participants selected notification times between 7:30 AM and 9:30 PM, with the mean time = 3:53 PM and the median time = 5:45 PM. The daily logs were submitted across all hours of the day, with the mean time = 4:15 PM and the median time = 6:22 PM.

For the structured symptom logs, participants self-tracked a range of symptom experiences. According to the “How was your day?” question, 5.6% of days logged were “Unbearable,” 17.8% of days were “Bad,” 39.4% of days were “Manageable,” 32.4% of days were “Good,” and 4.9% of days were “Great.” Individuals tracked between 0 and 16 symptoms on a given day, with an average of 7.1 (SD 3.8). For the activities of daily living that individuals found difficult to do, they tracked between 0 and 22, with an average of 7.6 (SD 6.6).

**Figure 2:**
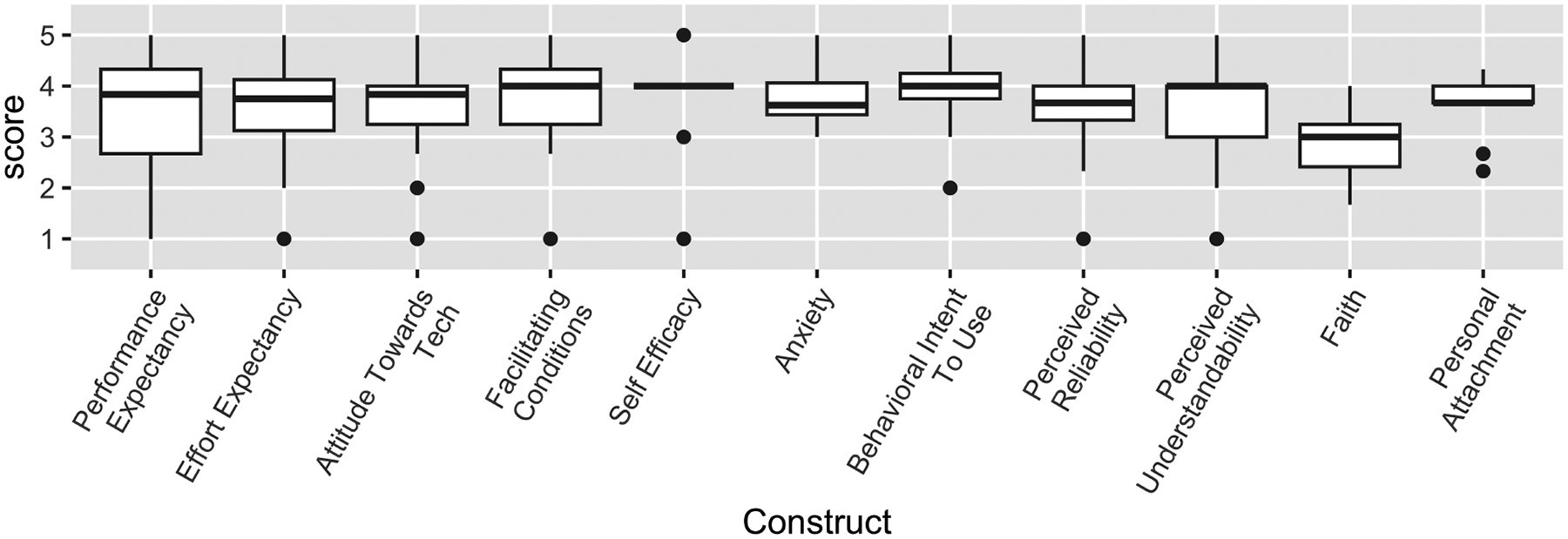
Technology Evaluation Measures. UTAUT constructs: *Performance Expectancy* - measures how well an individual believes that using the system will help complete the intended task; *Effort Expectancy (Ease of Use)* - measures how easy individuals believe the system is to use; *Attitude Toward Using Technology* - measures an individual’s affective reaction to using the system; *Facilitating Conditions* - measures the belief that they will be supported by technical and organizational infrastructure in using the system; *Self-Efficacy* and *Anxiety* - represent indirect determinants of intention to use the system; *Behavioral Intent to Use the System* - measures the ultimate intention of an individual to use the system. HCT constructs: *Perceived Reliability* - measures the belief that the system will perform consistently as expected; *Perceived Understandability* - measures the belief that a user understands how the system behaves and can predict its future behavior; *Faith* - measures the belief that they system will perform the task as intended; and *Personal Attachment* - measures an individual’s sense of attachment to the system, including finding the system agreeable and being partial to using it. Higher scores are more positive.

To assess how participants understood the purpose of the study, we asked them an open-ended question in the pre-study survey: “Can you briefly describe your understanding of why we are asking you to record your voice?” Participants described that the purpose of the research was to collect data to *“determine if a person’s voice markers can predict a flare-up”* and *“for user experience data”* related to the imagined tool we described to them. In the focus group, we elaborated on this speculative system before ideating together. Follow-up technology measures for the imagined intelligent system were promisingly high (Figure 2): Performance Expectancy, mean = 3.52 (SD 1.19); Effort Expectancy, mean = 3.55 (SD 1.11); Attitude Towards Technology, mean = 3.58 (SD 1.02); Facilitating Conditions, mean = 3.64 (SD 0.96); Self-Efficacy, mean = 3.81 (SD 0.91); Anxiety, mean = 3.78 (SD 0.64); Behavioral Intent To Use, mean = 3.88 (SD 0.96); Perceived Reliability, mean = 3.48 (SD 0.93); Perceived Understandability, mean = 3.55 (SD 1.11); Faith, mean = 2.86 (SD 0.64); and Personal Attachment, mean = 3.57 (SD 0.59). Overall, we find that participants were optimistic about using the system. This suggests that participants largely had a positive outlook on the potential for this technology to be useful to them in the care and management of their illness.

### 4.2 Themes

All individuals were invited to participate in the focus groups, and a total of n = 14 individuals were able to attend one of three focus groups. Discussions with participants affirmed benefits to self-tracking, and particularly using a voice modality, that have been previously documented in HCI. Individuals reported benefits in reflecting on their illness experiences and expressed that talking about their lives and endometriosis symptoms helped them to be more mindful about what they have been experiencing. Individuals also foresaw benefits to flare-up forecasts, which could help them organize their lives, prepare to engage in self-management, and engage in behavior changes to prevent or mitigate flare-ups. Beyond this, we found several novel insights that came up in this illness context that have not been widely documented. We generated three themes to describe these phenomenon. In particular, we note potential emotional benefits that individuals emphasized when talking about their experiences submitting voice logs, as well as some potential risks. We also note complex trade-offs discussed by participants as they imagined interacting with an intelligent system for forecasting flare-ups. Finally, we document the foreseen potential for voice logs and flare-up predictions to be valuable to users in validating their own illness experiences, and opportunities to use such technologies to communicate with their providers and validate their experiences with “objective evidence” in clinical settings.

#### 4.2.1 Logging health experiences with voice recordings has potential therapeutic and emotional effects.

While we instructed participants to talk about whatever they wanted in their recordings, all participants talked about their symptoms and the day-to-day impacts of their disease on their lives. In the focus group discussions, participants talked about how recording their experiences with their voice impacted these experiences and had potential emotional implications. Participants explained that recording their experiences with their voice was “intimate,” “grounding,” and “even more personal than answering the questions,” which they felt helped them to internalize their experiences. One participant explained that while the symptom checklist helped her to click through to understand her illness, the voice recording *helped me internalize and just talk about my feelings, and then focus on what I felt instead of what I thought I felt […] when you talk about it, you just have to remember, be in that memory, which makes you kind of feel again. [FG-C, Part. D]* Completing a daily check-in with the voice modality was a potentially useful mechanism to help individuals reflect on their experiences, understand their disease, and connect with the emotional aspects of their illness. Individuals appreciated the open-ended nature of the voice log instructions and most reported that they had enough to talk about, but participants across focus groups requested optional prompts to support their voice log submissions.

Participants emphasized that living with endometriosis can be a very frustrating and isolating experience due to its enigmatic nature and burdensome, systemic presentation and discussed what they described as emotional benefits to talking about their illness experiences in voice recordings. Participants talked about how creating the voice logs helped them to validate their negative feelings and how expressing their negative emotions helped to bring about relief. One participant described how cathartic it was for her to talk about how much she accomplished in a day, despite feeling unwell:

When I talked about my symptoms — ‘cause I was obsessed with my symptoms — I thought to myself, ‘You know, you did a lot today. You worked this many hours and you feel like crap and it’s not in your head.’ And so for me, it was very cathartic to talk about my feelings in a way that I never did.

[FG-C, Part. D]

Participants discussed that submitting voice recordings could potentially be healing for them. Rather than being a burden, submitting daily voice logs was perceived as an “outlet” where individuals could “vent” and “just get it out.” Several individuals even talked about, in a way, looking forward to having a flare-up so that they could take advantage of the non-judgmental space to talk about it in their recordings. One participant explained that it felt therapeutic to be able to talk about what was going on: *I was kind of hoping I would [have a flare], so I could be able to talk about it, because I knew I had a way to actually say it, get it out. [FG-A, Part. B]* Another person expressed that she was surprised that she was looking forward to submitting recordings when in a flare:

I thought flares would be a barrier, I thought there would be days where I would flare up and be like, ‘Oh, one more thing to add,’ but it actually ended up kind of being the opposite. It was like, if I was really flaring, I was kind of excited to record because I was like, ‘Oh, here’s my chance to talk about my symptoms’, and just get it out a little bit. So that was surprising to me.

[FG-A, Part. C]

Individuals discussed how they felt that reflecting on their experiences out loud on a daily basis helped them to calibrate how they are really feeling. As one participant described:

I also found it helpful just talking out loud. I realized that my definition of pain was almost accepting a certain baseline that I think would not be acceptable for most people. But, whenever my day good or manageable. I could get up, I could walk today, and I was like, ‘Is that really good?’

[FG-B, Part. B]

While many participants discussed the perceived positive impacts of logging their experiences with their voice, a few talked about the experience being neutral: *For me, it was fully neutral. It wasn’t aggravating or helpful. It was just reporting data. [FG-A, Part. A]* A few others discussed how they felt that recording their illness experiences with a voice log could bring up painful emotions or be frustrating to realize they are frequently in pain and experiencing a high burden of symptoms.

But going through flare, and checking in with yourself everyday, and recording your voice, talking about how your day went, I did feel frustrated just because I wasn’t feeling well for so many days in a row. And, here I am saying into this phone, ‘And five days later, I still feel really bad.’ But it’s not the experience of using it [to record my voice], it was just the experience of documenting that I am still not feeling well.

[FG-A, Part. D]

Another person described a similar sentiment, explaining that being able to dump her feelings into a non-threatening space helped her to sit with her experiences and make sense of her feelings, but at the same time was uncomfortable: *It did arise painful feelings, especially because I lost so much time. [FG-C, Part. D]*

#### 4.2.2 Flare-up forecasts have both imagined benefits and drawbacks.

With the constant threat of unpredictable flare-ups derailing the lives of individuals, participants imagined many potential benefits and several foreseen drawbacks to the possibility of an intelligent system forecasting upcoming symptom flare-ups. Many people talked about how forecasts of flare-ups could have the potential to help them to better plan and prepare for the future. They imagined various ways these forecasts might help with planning for work arrangements, vacations, and social situations. (*If you have the opportunity to reschedule some things and lighten up the schedule, then that would be really great. [FG-A, Part. D]*) All focus groups discussed the possibility of using flare-up forecasts to help plan for self-management and self-care ahead of flare-ups. They explained that in addition to helping prepare with self-management, having this information might help them communicate with their families, their jobs, and their friends. Focus group participants also speculated about the potential for forecasts of upcoming flare-ups helping them prevent or mitigate flare-ups by encouraging them to engage in positive lifestyle activities or avoiding negative ones.

For me, I know there’s certain foods that I eat that can make it worse. And even though I know that, I’ll still indulge in them sometimes. So maybe knowing that a flare is going to come, I would definitely try to not do that. Or I would try not to be as physically active or different things that I know put me particularly in pain, I could maybe try to stay away from.

[FG-B, Part. A]

Several participants imagined how forecasts of non-flare-ups might help them take advantage of times when they will feel good. (*If you know that tomorrow is not going to be very good, instead of putting off stuff to tomorrow. There’s that aspect that it’s like, ‘Oh, well, I might as well do it.’ [FG-B, Part. C]*) For example, one participant talked about how she could see the potential for an intelligent system to help her make an effort to connect with her daughter and foster other relationships during times when she was forecasted to have a relatively low symptom burden. She explained:

Maybe being able to know, ‘Okay, hey, in three days time, or at the end of the week, you’re probably gonna have a flare,’ I can make sure to really try to get quality time in with her before. […] And that doesn’t even have to apply to having kids, it can apply to really anyone in your life. But being able to really soak in those moments on the days that may be good before, when you’re not going to have those good days to come. Because I feel like a lot of us with this disease, I just watch my life just go by. And these relationships, they have to live their lives, and their lives just continue on. But you feel a bit left out.

[FG-B, Part. A]

Another person talked about potentially using forecasts of nonflare-ups to schedule a long travel day where she needed to feel well enough to drive for several hours. Instead of putting this drive off until another day when she may experience a flare-up, she envisioned using the forecasts to plan ahead and accomplish those tasks when she was feeling good. She said that the forecast might de-stress these situations, explaining: *Instead of putting things off, I’m hammering things out on to the day that I know, like, today I’ll feel good. So I’m just gonna do everything right now. Because I don’t know what tomorrow is gonna be like. [FG-B, Part. D]*

Beyond managing the physical symptoms of flare-ups and adjusting their day-to-day lives to accommodate their illness, participants also discussed the potential for forecasts to impact their anxiety around their condition. Several people commented that in addition to physically mitigating the burdens of flare-ups, forecasts might also alleviate some of the anxiety and stress of worrying that flare-ups are coming and help them feel in control of their health. As one participant explained: *I think being able to plan ahead would not only provide that physical relief of being able to care for myself, but it would probably relieve some anxiety of knowing, ‘Okay, I think I’m in pretty good shape today.’ [FG-A, Part. E]* Another person explained that even if there’s nothing she can do to prevent the flare-up, knowing that it is coming could potentially help her mentally prepare: *It’s inevitable, but I at least know, because I can also deal with the anxiety around that. [FG-A, Part. C]*

At the same time, several participants lamented that since there are no reliable treatment or management options for endometriosis, there is often nothing that can be done to prevent or mitigate symptoms so they worry that they would not experience any benefit to receiving forecasts. One person mentioned that she was unsure of what she would do with forecasts if she can’t change anything about the upcoming flare-up or circumstances around her.

It would be useful to know. But then the other side of it is what would I do with that information? Like if I know, tomorrow is going to be a bad day, but I don’t have flexibility within work? Or if you don’t have these other external things, then it’s like, do I need to know that?

[FG-B, Part. C]

Even further, a few participants discussed risks they foresee with flare-up forecasts, such as the potential for making symptoms feel worse or exacerbating anxiety around the flare. These negative effects risk making the experience of illness worse for some individuals. One participant explained that flare-up forecasts might increase her anxiety:

I wouldn’t want to know because I get very anxious, and it’s gonna ruin my nighttime, it’s gonna ruin my day. I’m going to be thinking about that all day, and I’m not going to be able to still accomplish things that I need to do because I’m thinking about this flare-up.

[FG-C, Part. B]

Several participants also discussed that the way forecasts might be presented and framed in a future system could potentially impact how they feel about receiving them. Being simply alerted to an upcoming flare was seen as potentially anxiety-provoking, while being prompted to engage in self-care was imagined as a way to reduce anxiety around a flare-up.

When asked about receiving flare-up forecasts from an intelligent system that will at least sometimes make incorrect forecasts, participants speculated that they would have a preference for false positives (forecasting a flare-up that does not happen) over false negatives (forecasting no flare-up when in reality symptoms do worsen). As one person explained:

I’d rather have it tell me, ‘I’m gonna have an awful day,’ and I feel good, versus telling me ‘I’m having a great day,’ and then I don’t. If it’s telling me I’m gonna have an awful day, I’m not going to cancel everything for the day. It is more just trying to manage those symptoms and get the mental preparedness, whereas it could be a little discouraging, and maybe make you lose trust in the system a little bit, if you feel terrible, and it says, ‘Nope, everything’s great, things are fine.’

[FG-A, Part. C]

There was broad consensus among participants that they would *be more frustrated if it was like, ‘You’re fine,’ and then I woke up and was flaring. [FG-B, Part. B]* Participants also imagined that too many incorrect forecasts, but especially too many false negatives, would diminish their trust in a system.

#### 4.2.3 Voice recording and analysis may have the potential to provide self-validation and support self-advocacy in clinical care.

Participants shared that individuals with endometriosis often have their experience of illness questioned (*Even though a giant mass was extracted from my body, there’s still gaslighting from my own self and from medical teams. [FG-A, Part. D]*), and even end up questioning their own experiences (*You just feel like, ‘Is this all in my head?’ Or, ‘Is this real?’ [FG-A, Part. E]*). Many individuals talked about how engaging in talking about their health experiences out loud felt validating, and how analyses of their voice data could hypothetically provide a biometric signal that might affirm their symptom experiences. As one participant explained: *It was good to talk about your symptoms and how you’re feeling, and it just reaffirms that it’s real, and it is happening. [FG-B, Part. C]* Participants emphasized that in the context of endometriosis, information based on more “objective” data such as vocal characteristics could potentially help them combat doubting themselves and their experiences of illness. As one participant said: *Any validation, reassurance, clarification, anything that tells me this is not all in my head, there’s real evidence, is helpful for me. […] I will take as much help with getting an objective, real footing in what’s happening as possible. [FG-A, Part. D]* Another person imagined how this validation could counteract invalidating experiences:

Having the data that validates the internal mysteries that are going on is really helpful. I mean, I think we’ve all felt crazy at points, dealing with these things. And so it would it would just, every time you’re validated, it erases that one appointment that they didn’t believe you.

[FG-C, Part. A]

Many participants described experiences of being “brushed off” and “dismissed” by providers and feeling like their illness is “all in my head” or questioning if their experiences of symptoms are even “real.” So having logs of themselves talking about their experiences day-to-day was seen as providing reassurance that “there’s real evidence” of their disease, combating the lack of visibility of their pain.

Hearing myself saying the same thing everyday, reinforced it to myself. Because as well, there’s dismissal by doctors and this and that. So sometimes I think I can find myself thinking, ‘Oh, no, I’m fine, it’s probably nothing blah, blah, blah.’ But then having that every day, being able to actually monitor how I’m feeling, I thought was quite helpful in a similar way that it was like, yeah, actually, no, this is quite serious. I do feel and say quite a lot for the time. So I think that aspect of it was quite helpful and it was just a good thing to do to, kind of like, build your own word or how you’re feeling in a different way.

[FG-B, Part. C]

Individuals across focus groups talked about how reassuring it could be to have objective data, built up from the personal and intimate voice logs, to know their disease is real and they are “not making this all up” and “it’s not just my emotional state or my mental state.” When asked about what data they want the hypothetical AI to use, participants overwhelmingly imagine a system that can use both the biometric signals from their vocal characteristics and also the relevant content of what they said in their logs. However, while some participants wanted to be able to view and search their transcripts, none could foresee wanting to have access to listen to their voice logs after they were submitted.

With the stigma around endometriosis, its enigmatic nature, and patients’ constant struggle to be believed, logs could also have the potential to support individuals in their clinical encounters. Beyond providing validation for themselves that what they are experiencing is real and not in their heads, several participants discussed ways that they foresee logging their experiences could help them validate their experiences with their providers and communicate with their care teams.

Having the data to help for your own records and validation, and particularly if the app can provide some data that you can use with providers to show that you’re not crazy, and these are real symptoms, real things happening, that would be great.

[FG-A, Part. D]

The logs and results of analysis of their vocal characteristics (both future-oriented forecasts and detection of flare-up status in the past or current time-frame, without future predictions) were envisioned as pieces of “objective evidence” that they might use to help support them in seeking care and prove that their experiences are real. For example, one person explained that an intelligent system that enabled them to log their symptoms and that might provide an analysis of flare-ups could potentially help them *keep an eye on my pain flare-up, so I could explain to my doctors, ‘Hey, I’m having this pain flare-up. Not because I’m bothering you all, but because it is real.’ [FG-B, Part. E]* Another participant elaborated on this potential benefit, saying that this evidence of their illness experience *helps the doctor to understand that this is not spur of the moment, this has been going on every day. [FG-C, Part. D]* Participants discussed that their data could potentially help mitigate the mistrust between patients and providers and help them feel heard. While a real-word system capable of analyzing voice was not deployed for this study, participants experienced a variety of benefits to logging their voice and imagined an array of hypothetical benefits to such an imagined system.

## 5 Discussion

This research seeks to address the needs of people with complex chronic illness, by investigating the voice as a potential low-burden modality for capturing the illness experience and imagining this data as a possible biometric, speculating about using these signals to forecast symptom flare-ups to guide health actions. In this study, we engaged individuals with endometriosis in a speculative design activity over a 6-week period and then brought together individuals to discuss their experiences in collaborative, generative reflection sessions. Findings from this study suggest that an intelligent system for recording illness experiences and forecasting symptom flare-ups could possibly enable individuals to enact control over their illness narratives, their health, and their day-to-day lives. Using a voice modality could mitigate the demands of self-tracking while providing possible added benefits to capture nuanced physiological data, while potentially addressing emotional needs of living with a burdensome chronic illness. Results also suggest that such a system could also provide validation for individuals, which might also enhance autonomy and control — it is important to be believed. Feeling in control of one’s life is empowering. There are also imagined potential benefits to offering forecasts of symptom flare-ups, but findings also highlighted foreseen possible risks that must be considered when designing such an intelligent system. In this section, we discuss the broader implications of this work, connect findings to key principles of human-centered AI, and unpack critical aspects to consider in moving this work forward.

### 5.1 Potential benefits and opportunities for data capture with the voice

Using the voice to document the experience of illness allowed individuals in this study to collect personal health data while potentially reducing the burden of tracking and opening up the possibility of providing a source of “objective” data on health status. Participants in this study also imagined voice logs as a valuable tool to help create meaning and manage emotions associated with their illness. Echoing prior research [82, 84], conversations with participants in this study suggest that voice-based tracking might provide a flexible, dynamic, and deeply personal mechanism of documentation compared to traditional symptom checklists. Considering these imagined benefits of voice logging, even if a future personal informatics system does not include the functionalities to forecast symptom flare-ups, users may still benefit from a feature that allows them to log their illness experiences in voice recordings.

Others have investigated the benefits of multimodal tracking, including using voice, and using these data to create meaning. For example, Silva et al. found that integrating voice, images, and text across devices in food journaling improved accuracy, ease, and enjoyment, enabling richer, more contextual data capture and personalized insights [98]. Similarly, Raj et al. proposed “episode-driven narratives” to help patients with diabetes make sense of multidimensional health data, empowering them to identify patterns, gain insights, and make informed decisions [89]. Strömel et al. provide an example of using large language models to translate complex fitness tracker data into meaningful narratives, fostering deeper reflection and understanding [101].

In this study, we find that the voice might function as an instrument of mindfulness, intentionality, and introspection regarding the embodied experience of illness, aligned with prior work [66]. Discussions with participants suggest that using voice has the potential to change and enrich the illness experience by facilitating personal reflection, helping individuals craft and validate their narratives, process their emotions, and feel empowered in their illness journeys and in their encounters with care teams. These findings represent a potential meaningful and constructive exchange between the human and technical components of such a technology. However, while we found that participants expressed potential emotional benefits to logging their experiences with their voice, Eikey et al. warns that excessive reflection on these data, or rumination, can induce anxiety and over-analysis of emotional triggers [30]. We note that the potential emotional benefit was discussed about the act of recording, rather than using voice as a journal to be re-visited in the future. The fact that participants in this speculative study did not want access to their recorded sessions suggests that the sole act of expressing their emotions and illness experiences could hold therapeutic value, even without the presence of a listener. This is not necessarily supported by prior work, which emphasizes the value in retrospective reflection [55].

### 5.2 Imagined utility of forecasting

Through conversations with participants, we uncovered a tension in the potential application of a predictive technology for forecasting symptom flare-ups. On one hand, participants in this study imagined that flare-up forecasts have the potential to help individuals structure their lives, prepare for or mitigate flare-ups, take advantage of good days, and manage the anxiety and uncertainty around their complex chronic illness. On the other hand, they also expressed concerns that these forecasts have the potential to make their flare-ups feel worse, amplify frustration when flare-ups seem unavoidable, or exacerbate anxiety about their illness.

Others have found similar potential benefits and risks that we documented in our study. Rho et al. introduced a tool that visualizes future health statuses based on self-tracking data, encouraging users to reflect on their lifestyle choices and how they could adjust their behaviors to improve future outcomes. Although the tool motivated users to change their behaviors to prevent negative health events, some experienced anxiety and pressure when they felt powerless to avoid negative outcomes [90]. Similarly, Katz et al. documented negative practical and emotional impacts of interacting with personal informatics systems, suggesting potential paths forward for designing tools that address the challenges of engaging with “bad” data representing undesirable states [56]. Future-oriented work in diabetes management also highlights these complexities — Barth et al. documented both risks and opportunities, showing that while forecasts could trigger emotional distress, they could also support personalized insights and sensemaking [8]. Desai et al. similarly found that forecasting could aid reflection and guide actions [25]. In the domain of mental health, several studies have documented benefits of forecasting and provide guidance for design. Hollis et al. developed *EmotiCal*, a forecasting system that uses past mood data to model and visualize future states, promoting reflection and action. They found that engaging with positive past data was beneficial, but warned that revisiting strongly negative experiences may lead to distress [48]. Tateyama et al. presented users with future forecasts to help them plan and regulate their health status, showing that forecasts helped users proactively plan and gain a big-picture view on their health. However, users sometimes felt constrained by the forecasts and felt stressed by the perception that negative outcomes were inevitable [102]. These findings mirror both imagined benefits and also concerns brought up in our study, highlighting the importance of balancing these foreseen risks and benefits of forecasting technologies for chronic illness management.

### 5.3 Speculative design considerations grounded in human-centered AI for chronic illness management

In human-centered AI, humans are positioned at the core of the development life cycle of intelligent systems [14]. It is critical to center the needs of users, and in particular to engage them as experts of their own experiences and data, which is how we have approached this research. Human-centered AI tools have the potential to empower human users and should strive for high levels of human control alongside the automation that AI can provide [96]. Various reports from professional societies, government agencies, consumer groups, and corporations have identified a wide range of key principles that characterize human-centered intelligent systems [21, 35]. In this section, we discuss how our findings fit into some of these critical human-centered principles.

#### Autonomy and Control.

Findings from this speculative design study suggest that personal informatics systems that leverage the voice for capturing illness data might have the potential to empower individuals by helping them feel in control of their lives and illness narratives. Having evidence, whether in the form of transcripts from voice logs or outputs from flare-up analysis, to substantiate lived experiences might help individuals feel validated and further enhance feelings of autonomy, agency, and empowerment.

Shneiderman emphasizes that truly human-centered AI systems must enable meaningful human control, even when a system is powerful in its AI capabilities [96]. Building on this principle, we propose several design implications for creating human-centered technologies. These recommendations aim to enhance user autonomy and control while maximizing the potential benefits and mitigating possible risks of a voice-enabled intelligent system.

- **Voice characteristics vs content.** Allow users to choose whether the system analyzes only voice characteristics (e.g., tone, pitch, or cadence) or also includes the content of their recordings. This allows users to control the level of privacy and data shared for analysis.
- **Optional prompts.** Allow users to freely express them selves in voice recordings while offering optional prompts for those who may feel unsure about what to talk about. These prompts can guide individuals and help reduce the burden of submitting voice logs, while providing freedom to talk about whatever feels important.
- **System output.** Provide flexible options for using the system, allowing users to tailor their interaction with the system and indicate what, if anything, they want to receive as output. Some users may prefer to use a voice recording feature as a private journal without receiving any feedback; alternatively, users may want to receive output from analysis of their voice data to assess and monitor flare-ups (i.e., detection of past and current health status), but do not want future-oriented forecasts; further, users may want to receive future-oriented health status forecasts, but still may want control over which ones to receive (e.g., under what level of certainty, or only forecasts of non-flare-up days) and how to receive them (e.g., as simple alerts vs personalized suggestions to engage in self-care behaviors).

#### Trust.

There are inherent limitations to AI and its ability to forecast adverse outcomes such as flare-ups. Therefore, designers must prioritize transparent communication about the performance of forecasting models, presenting this information in ways that are easily understandable by lay individuals. Collaborating with end-users will be essential to ensure that systems center their expertise and align with their needs and expectations. Research highlights both opportunities and challenges in fostering trust in AI. Hollis et al. documented that people are willing to trust an intelligent system with intimate aspects of their personal self and associated risks with taking system outputs at objective face-value [49]. Over-reliance on AI has been shown to lead to incorrect actions, underscoring the need for caution in design [19, 53]. Liao and Sundar emphasize the importance of empowering users to make informed trust judgments by designing mechanisms that clarify the trustworthiness of a system [63]. Such strategies can help balance trust and skepticism, supporting effective and responsible use of AI systems.

#### Privacy.

Privacy is of high importance in human-centered AI systems [81]. In a system for capturing and analyzing voice logs about illness experiences, there are significant implications for the privacy of such intimate data. In this context, privacy considerations become even more critical due to the deeply personal, emotional, and vulnerable nature of the recordings, which reflect participants’ self-expression and lived experiences.

Participants in this study expressed a desire for intelligent systems to analyze not only the vocal characteristics of their logs but also the content, raising additional privacy implications (e.g., users may not have a secure place to talk about their illness out loud without being overheard). This concern is heightened in the case of endometriosis, a stigmatized condition where patients often face dismissal and disbelief from healthcare providers — a challenge also documented in research on women’s menstrual and reproductive health [41]. Even the act of capturing voice data presents privacy risks, particularly given the identifiable nature of voice recordings. Designers must consider ways to enable individuals to document their experiences while safeguarding their privacy, e.g., allowing users to record logs on broader topics, rather than limiting input to illness-specific details. Ensuring robust privacy protections for data collection, storage, and usage is therefore essential for fostering trust and supporting users.

## 6 Study Limitations and Ethical Concerns

This study has several limitations. First, while the use of a speculative design approach allows for creative ideation and accounting for human-centered perspectives early in the design process, it also has some inherent limitations to consider [29, 39]. It relies on users to imagine a technology that might suit their needs, but results are open to (mis)interpretation, and it cannot be confirmed that stated desires are really what users want. In this way, this design approach may not actually be reflective of user behavior or attitudes once the imagined design has been created. Further, while it expands possibilities and promotes innovation, it may not be ethical, practical, or even possible to create the technologies that individuals imagine, since participants are asked to be as creative and speculative as they want. In this case, no forecasts were given and individuals were asked to imagine that they could receive a forecast of an upcoming flare-up — such a tool is not yet developed and its accuracy has not been tested.

There are also some limits to our study specifically. We relied on Qualtrics for data capture on participants’ phones and not an app specifically designed for our envisioned functionality. Further, in setting up the notifications, we encountered technical trouble that caused temporary delays in the delivery of notifications (i.e., the automated process was terminated and needed to be restarted), which did not impact the ability to submit daily logs. This would be resolved by deploying an actual app in future studies. This study is also limited by the representativeness of the sample, especially because we recruited individuals already engaged in self-tracking their disease.

Finally, there are various ethical concerns to take into account in studying the potential use of voice for forecasting symptom experiences and in the design of an intelligent system that leverages voice. Voice data are inherently personal, and analyses could reveal sensitive information about a person’s health, emotions, or identity. There is an additional risk with the potential for data breaches, which could result in compromised sensitive information. There are also limitations in analysis capabilities that risk an over-reliance on results, which could mislead users; this is exacerbated by the “black box” nature of AI, making it difficult for users to understand how predictions are made, and this lack of explainability can undermine trust of the system. Voice capture and analysis could also feel invasive, and especially if outputs reveal certain health states, these results could lead to increased stigma or discrimination in personal or professional life. Further, all AI models have the potential to inherit or learn biases from training data, leading to inaccurate or inequitable predictions across demographics (e.g., accent, age, gender, ethnicity), which could result in unfair treatment or mis-classification that disproportionately affects vulnerable populations. Finally, these tools could be repurposed for surveillance or oppressive purposes, such as monitoring vulnerable populations. Mitigation strategies include ensuring transparency in data practices, using diverse datasets to train models, adopting bias audits, enabling user control over data, incorporating human oversight, and conducting robust ethical reviews.

## 7 Conclusions and Future Work

This study reports on a promising direction for supporting individualized management of chronic illness with a personal informatics tool. We speculate about a system that might leverage low-burden voice logs to forecast illness flare-ups. It remains unclear if voice characteristics and speech patterns might be suitable to provide signals of underlying physiological phenomenon or health status, and future research — e.g., with the voice logs collected in this study — will be needed to investigate the potential of voice as a biometric for systemic inflammation. Nevertheless, we discuss real-world user perspectives on an imagined tool and provide key insights for designing such an interactive system in the future.

## Supplementary Material

supplementary material

## Figures and Tables

**Figure 1:**
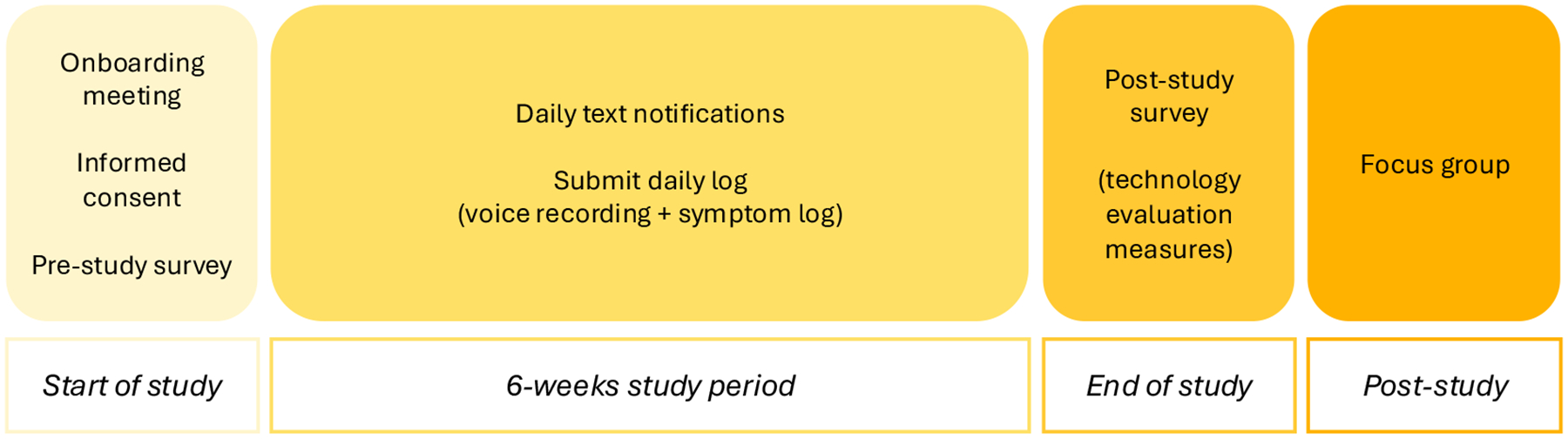
Study Overview.

**Table 1:** Sample Data (N = 21).

(a) Participant demographics.	
**Age**	
Mean (SD)	33 (8.1)
Median	31
Range	20–50
**Gender**	**n (%)**
Woman or Female	20 (95)
Non-Binary or Genderqueer	1 (5)
**Race**	**n (%)**
Black	3 (14)
Hispanic	5 (24)
White	14 (67)
**Relationship Status**	**n (%)**
Married or domestic relationship	11 (52)
Single, never married	10 (48)
**Highest Level of Education**	**n (%)**
College +	16 (76)
Some college	5 (24)
**Income**	
Mean (SD)	80k (71k)
Median	70k
Range	0–300k
**Employment Status**	**n (%)**
Employed	15 (71)
Not employed	3 (14)
Student	3 (14)
**Living Environment**	**n (%)**
Rural	1 (5)
Suburban	9 (43)
Urban	11 (52)
(b) Participant menstrual characteristics.	
**Frequency of pelvic/abdominal pain, past 3 months**	**n (%)**
Every day	7 (33)
More than one day a week	7 (33)
One day a week	3 (14)
Two to three days a month	2 (10)
Did not have pain	2 (10)
**Any periods, past 3 months**	**n (%)**
Yes	15 (71)
**Pain level during last period**	**n (%)**
Mild cramps	3 (14)
Moderate cramps	7 (33)
Severe cramps	11 (52)
**Pelvic pain interfered with work, school, or carrying out daily activities, during last period**	**n (%)**
Yes	16 (74)
**Need to lay down during the day because of pelvic pain, during last period**	**n (%)**
Yes	20 (95)
**Severity of pain during last period (1 least severe, 10 most severe)**	
Mean (SD)	7 (2.2)
Median	8
Range	1–10
**Frequency of pelvic pain with periods, past 12 months**	**n (%)**
Always (every period)	14 (67)
Usually (more than half of my periods)	3 (14)
Often (a quarter to half of my periods)	3 (14)
Never	1 (5)

Note: Participants could select more than one race/ethnicity; race and ethnicity were asked together. Categories with no responses have been omitted from the table (Asian, Native American, Other).

## CCS Concepts

• **Human-centered computing** → **Interactive systems and tools**; **Smartphones**; • **Applied computing** → **Health informatics**.
