# Supplementary material for: The Voice of Endo: Leveraging Speech for an Intelligent System That Can Forecast Illness Flare-ups
Source: Proc SIGCHI Conf Hum Factor Comput Syst. Author manuscript; Available in PMC 2025 Sep 17. (PMC12439622; doi:10.1145/3706598.3714040)
Supplement: supplementary material [file NIHMS2074952-supplement-supplementary_material.pdf]

1 SUPPLEMENTARY MATERIAL

1.1 Onboarding Session

- Welcome, and thanks for joining the session!
- Brief introduction to the research team and broader research projects
- Overview of the research study
  - Exploratory study to see: can voice predict an endometriosis flare-up?
    We are recruiting participants to help us explore whether it is possible to predict an endo flare from your voice. Research shows, for instance, that the tone of someone's voice is a promising marker for stress or even inflammation.
  - Two parts:
    (1) Collect data (from the daily log) for analysis and machine learning, to assess the feasibility of a tool (is it possible?). We ask — can different voice characteristics (e.g., tone, pitch) be informative markers for the types of symptoms people experience, and inflammation?
    (2) Assess user experience and perspectives (via focus groups) on the proposed tool for using voice recordings to receive health insights. We want to see whether individuals living with endometriosis will benefit from such a technology in a safe and ethical way.
- Details on this research study, what is being asked of participants, and how participants will be compensated
- Confirm eligibility criteria
- Informed consent process, and answer any questions
- Get set up with the study
  - Ask user what time to set up Notification (reminder to submit daily log)
  - Give instructions for submitting the daily log
    - Record yourself for 1-2 minutes every day using the microphone in your iPhone
    - Talk about whatever you choose (reflect on the day, symptoms, activities, work, personal life).
      Speak naturally, without a script!
    - Minimize background noise and speak clearly
    - Try to record at the same time every day
    - Upload the unedited recording via the personalized Qualtrics link
- Instructions for the next 6 weeks
  - (Today) Please complete the baseline survey that has been emailed to you
  - (Daily) You will receive a text with a link to the daily log survey, where you will record and upload a voice recording and report your symptoms
    - You are encuraged to complete the log as often as possible
    - If we notice you are not active in submitting daily logs for over a week, we will reach out to you to check in!
  - (At the end of the 6-week study)
    - We will send you the follow-up survey to complete
    - We will also schedule remote focus groups
- Thank you! Here are emails if you have any questions or issues, please reach out.

1.2 Daily Log Survey

Page 1: Submit voice recording

Q1: Tell us about your day. *(Capture or upload voice file)*

Q2: Notes or details about voice recording for today. *(optional text entry)*

Page 2: Daily check-in questions

Q3: How has your day been?

- Great
- Good
- Manageable
- Bad
- Unbearable

Q4: Did you have your period today? If you do not have a period, you can leave this question blank.

- Yes
- No

Q5: Since you woke up today, in which of these body locations have you experienced pain? *(check all that apply)*

- Pelvis
- Uterus, Cervix, Ovary
- Vagina
- Lower Back
- Abdomen, Intestines
- Lower Body
- Upper Body
- Head (including headache), Neck
- Bones, Joints
- Rectum

Q6: Since you woke up today, which of these symptoms have you experienced? *(check all that apply)*

- Fatigue
- Endo Belly or Bloating
- Nausea or Vomiting
- Brain Fog

Q7: Since you woke up today, which of the following activities were hard to do (or would be hard if you tried)? *(check all that apply)*

- Sleep
- Get out of bed
- Stretch
- Use toilet
- Take a shower or bath
- Get dressed
- Prepare food
- Eat
- Work
- Socialize
- Have sex
- Shop
- Housework
- Sit down
- Lie down
- Stand
- Walk
- Run
- Jump
- Kneel
- Lift
- Climb stairs

Q8: How often have you been bothered by the following problems today? *(select one option for each row)*

	Not at all	A little of the day	A lot of the day	Most of the day
Q8a: Feeling nervous, anxious, or on edge				
Q8b: Not being able to stop or control worrying				
Q8c: Little interest or pleasure in doing things				
Q8d: Feeling down, depressed, or hopeless				

1.3 Focus Group Guide

- Hi everyone, and welcome to the focus group! Thank you for contributing to this research study; we appreciate your participation in the focus group today! We are interested in your experiences and value your opinions.
- Today's discussion will focus on hearing about your experiences in the Voice study – both practical aspects, and how you might imagine using a tool if it existed within a self-tracking app. This information from today's session will help us design tools that respond to the needs of patients such as yourselves.
- You've already signed the consent form at the start of the study, but we just want to remind you about a few things... The information you share is confidential, and we will not associate your identity with anything you say in the focus group.
- We understand how important it is that this information is kept private and confidential, particularly because we will be discussing personal health issues. We ask participants to respect each other's confidentiality. Therefore, we ask that no information about participants should be shared outside of this group. You can also refuse to answer any question, and if it's too difficult for you to talk about, we understand if you need to step away or leave the focus group.
- We will be recording the discussion so that we can make sure to accurately capture the thoughts, opinions, and ideas from the group. We will also take notes. No identifying information will be attached to the focus group tapes or notes.
- We will use the information gathered here to inform the design of new tools for managing endometriosis. In addition, we plan to submit a research paper describing the main insights gained from this entire study for publication in an academic journal.
- The focus group will last approximately an hour and a half.
- For compensation, you will all receive $25 for participating in today's group. After the session, your total compensation from all study activities will be calculated and then mailed to the address we have on file. Please email us with an updated mailing address, if you need to!
- Have folks participated in focus groups before? *(Wait for people to respond).*
- We are here to learn from you! We want to know about both positive and negative opinions. The point of our work today is to gather information. We are not trying to achieve consensus; therefore, there are no right or wrong answers to our questions. Especially when it comes to endometriosis, there is a very wide range in the way people experience it – so again, no right or wrong way to answer.
- In order to make sure the focus group goes smoothly, we want to lay out some ground rules. If you would like to add or change anything please speak up and we can amend.
  - Everyone should participate – I may ask for particular people to share if they have been quiet o Do not share information you hear outside of this group today to respect everyone's privacy (We've changed everyone's names to first names also to respect privacy)
  - Try to sit in a quiet, private space and turn off cell phones or other distractions, if possible
  - We encourage everyone to have their cameras on; mute yourself if the background is noisy, but unmute to talk!
  - Have fun! Our chat shouldn't be TOO formal
- Are there any questions before we begin? *(brief pause)* If you have any questions now or later, you can reach out to any of the facilitators. Great, let's start the recording and get started!

First, we can introduce ourselves – let's go around the room and share our names and a little bit about your endometriosis experience – how long you've had symptoms for, how long you've been diagnosed. If you have an (un)fun fact about endo, please share! We will hear a lot about your experiences throughout the focus group today, so just give us 3-4 sentences!

Discussion begins. Give people time to think before answering the questions and don't move too quickly. Use the probes to make sure that all issues are addressed, but move on when starting to hear repetitive information.

A. Our first set of questions relate to your experiences recording your voice for the study. We are less interested in the technical aspects of you recording your voice, but we are very much interested in your experiences and reasons why you were or were not excited about recording yourself. When you answer, think of your every day lives, whether with an endo flare up or without, and how the experience of recording worked out for you. *(40min)*

(1) What did you end up talking about? (we don't need details, but more like themes)
  (a) Did it change from day to day?
  (b) Did it change when experiencing flare ups or difficult endo days?
(2) Where and when did you find yourself recording your voice?
  (a) Think about the entire duration of the study, did you change where and when you record yourself
  (b) Think about when you had more or less symptoms, did it change based on your disease?
  (c) Was there anything about your routine that prompted or helped to record your voice?
    - Did the daily notifications help remind you to record yourself?
(3) What barriers did you encounter to recording your voice?
  (a) What prevented or dissuaded you from recording yourself (emotions/motivation, outside circumstances, remembering to record, technical difficulties, interference with daily routine)?
    - Did it change day to day, or during disease fluctuations?
  (b) If you felt barriers, which ones were unexpected to you?
  (c) If you had a preconceived idea of what would dissuade you from recording, were you right?
(4) How did you feel about the overall experience of recording your voice ?
  (a) What feelings or emotions came up as you recorded your voice?
  (b) Were there any benefits you experienced from recording your voice?
  (c) Were there any unintended consequences or anything surprising that happened as a result of recording yourself?
    - Was it difficult talking about your disease? Was it useful?
  (d) Did you feel you had enough to talk about?

B. Next, imagine that we are building an app, and it uses AI so that it can detect, based on some data, that you are very likely to have a bad endo day in the near future. *(10 min)*

(5) *(utility)* In previous focus groups, we learned from patients like you that it would be useful to know when a flare up is coming. Can you tell us under which circumstances this would be useful? **Think independently of whether the voice recordings would be used for this.**

(a) How many days in advance would be ideal for you to know? One way to think about it, is would it give you enough time to try to prevent it or organize your schedule?

(b) AI tools are not always correct in their predictions. Under which level of certainty would you want to be notified of a flare up – would you rather it always predicts flares but get some wrong, or miss some but is always right?

C. Now, let's think about the fact that the AI will use your voice and the content of your recording to predict these bad endo days. For the rest of the discussion time, we want you to think about how the voice recordings could be useful to you. Imagine that we are building an app, where you can record yourself, and it uses AI so that it can detect that given the quality your voice or what you say during the recording you are very likely to have a bad endo day in the near future. *(30 min)*

(6) *(functionalities)* What kind of functionalities would you want the app to provide for this? (can be creative to imagine)

(a) *(interpretability)* Would you like a score? What else about the prediction

(b) *(explainability)* How much detail about why?

(c) *(feasibility)* Was it realistic to record for 1 minute? Too long? Too short?

(d) *(feasibility)* How often would you be willing to record for? In the study we asked you to do this daily, but for you what would be ideal?

(7) Would you? Why would you use this app? How might it change your experience of endo?

(a) How and what would you use these AI predictions for?

- Independent self-management, communicating with providers)?
- When you start feeling like a flare up is coming up? All the time?
- What if the output of the system differs from how you are feeling?

(b) How would you feel if we discovered that your voice correlates with other self-tracked information about yourself (e.g., inflammation, pain, menstrual cycle)?

(8) *(privacy and data ownership)* Now, let's think about the fact that the AI will use your voice and the content of your recording. This is an AI that is within the app, and all your information stays confidential and secure.

(a) Are you comfortable with recording yourself and talking about your daily life and experience of endo or anything else?

(b) Are you comfortable with an AI system that analyzes your voice but doesn't understand what you are saying, or would you be ok with an AI that also analyzes what you talk about?

(c) This is your data and so you own it, but would you want to have access to it? Why?

(9) Any other worries or good things about this app that would make you not use or tell your endo friends about it?
